# The association between nonalcoholic fatty liver disease and esophageal, stomach, or colorectal cancer: National population-based cohort study

**DOI:** 10.1371/journal.pone.0226351

**Published:** 2020-01-24

**Authors:** Jung-Min Lee, Yong-Moon Park, Jae-Seung Yun, Yu-Bae Ahn, Kang-Moon Lee, Dae Bum Kim, Ji Min Lee, Kyungdo Han, Seung-Hyun Ko

**Affiliations:** 1 Division of Endocrinology and Metabolism, Department of Internal Medicine, College of Medicine, The Catholic University of Korea, Seoul, Korea; 2 Epidemiology Branch, National Institute of Environmental Health Sciences, National Institutes of Health, Research Triangle Park, NC, United States of America; 3 Division of Gastroenterology, Department of Internal Medicine, College of Medicine, The Catholic University of Korea, Seoul, Korea; 4 Department of Biostatistics, College of Medicine, The Catholic University of Korea, Seoul, Korea; Kaohsiung Medical University Chung Ho Memorial Hospital, TAIWAN

## Abstract

We investigated the association between nonalcoholic fatty liver disease (NAFLD) and gastrointestinal tract cancer in the general population. Retrospective data on individuals aged ≥20 years who received healthcare checkups from January 1, 2009 to December 31, 2009 were analyzed using the National Health Insurance Database in Korea. NAFLD was defined based on the fatty liver index (FLI ≥60). The primary outcome was newly diagnosed esophageal, stomach, or colorectal cancer using ICD-10 codes during follow-up until 31 December 2017. Cox regression analysis was used to estimate hazard ratios (HRs) and 95% confidence intervals (95% CIs). Among 8,120,674 subjects, 936,159 adults (11.5%) were identified as having NAFLD. Their mean age was 46.7 ± 14.1 years, and 52.1% were male. During the follow-up period (7.2 years), 3,792 esophageal, 57,292 stomach and 68,769 colorectal cancer cases were identified. FLI ≥60 was significantly associated with the development of esophageal (HR 2.10, 95% CI 1.88–2.35), stomach (HR 1.18, 95% CI 1.14–1.22), and colon cancer (HR, 1.23, 95% CI 1.19–1.26) after multivariable adjustment. Compared to subjects without NAFLD, all-cause mortality in patients with esophageal (HR 1.46, 95% CI 1.28–1.67), stomach (HR 1.26, 95% CI 1.18–1.34), and colorectal cancer (HR 1.16, 95% CI 1.10–1.22) was significantly increased in subjects with NAFLD (FLI ≥60). NAFLD defined using FLI was a good predictive indicator for GI tract malignancy and all-cause mortality in the general population. Subjects with NAFLD are needed for active surveillance of esophageal, stomach, and colorectal cancers.

## Introduction

Throughout the world, cancer has been the most common cause of death. In particular, the Asian population has a higher incidence of gastrointestinal tract cancer than Western countries [1]. In 2012, the incidence of stomach and colorectal cancer in Korea was especially high worldwide, with an age-standardized rate of 41.8 and 45.0 per 100,000 persons, respectively [2].

Nonalcoholic fatty liver disease (NAFLD) can be defined as the presence of greater-than-normal lipid accumulation in the liver without excessive alcohol consumption [3]. With an increase in Westernized lifestyle, the prevalence of NAFLD in the Asian population has steadily increased in recent years [4]. NAFLD is closely related to chronic metabolic diseases such as obesity, insulin resistance, and type 2 diabetes and is one of the most prevalent chronic liver diseases, at approximately 20–40% of the general population [5–7]. The prevalence of NAFLD in the general population has been reported as 11~45% and 8~42% in North America and Asia, respectively [8,9]. In a cross-sectional study of 140,000 Korean participants in a health screening program, the NAFLD prevalence rate was reported as 25.2% [10]. Without treatment, 10–29% of patients with NAFLD develop cirrhosis within 10 years [11]. In addition, NAFLD can progress to liver cirrhosis and hepatocellular carcinoma [11,12]. Fortunately, some risk factors for the development of NAFLD are known; therefore, active intervention such as lifestyle modification, reduction of body weight, and some medications might be helpful for the progression of NAFLD after diagnosis [13].

Because of the pathogenic factors of NAFLD, including insulin resistance and abdominal obesity, that could influence colorectal neoplasm development [14], several studies have investigated the association between NAFLD and colorectal neoplasms [14,15]. NAFLD (diagnosed by imaging study) independently increased the risk of overall colorectal neoplasm occurrence and severity at the time of the surveillance colonoscopy [16–18]. In a retrospective study in Chinese females, combined NAFLD and metabolic syndrome was an independent risk factor for colorectal cancer-specific mortality [19]. In addition, one meta-analysis suggested that NAFLD may increase the risk of cholangiocarcinoma, with a pooled odds ratio of 1.95 (95% confidence interval (CI): 1.36–2.79) [20]. However, the association between NAFLD and other extrahepatic malignancies, especially gastrointestinal tract cancer, has not been fully investigated.

The fatty liver index (FLI), which is an algorithm based on waist circumference (WC), body mass index (BMI), triglycerides, and gamma-glutamyl-transferase (GGT), was initially developed to detect fatty liver in Western countries [21]. It has been validated as a practical, reliable, and noninvasive method to diagnose NAFLD in large epidemiologic studies, including the Asian population [22,23]. European NAFLD guidelines recommend serum biomarkers and scores as an acceptable alternative for the diagnosis of hepatic steatosis in the general population [24]. According to one retrospective observational study in a relatively healthy Asian population, subjects with a high FLI had a higher prevalence of colorectal adenomas [25].

Therefore, we aimed to evaluate whether FLI, a noninvasive, simple predictor of NAFLD, was associated with the development of gastrointestinal (GI) tract cancer, focused on esophageal, stomach and colorectal cancers, in the general adult population using the Korean National Health Insurance Service (NHIS) claim database.

## Materials and methods

### Source of the database

In this retrospective cohort study, we used the NHI database maintained by the Korean NHIS, a government-affiliated agency under the Korean Ministry of Health and Welfare that supervises all medical services in Korea. All Korean subscribers are encouraged to receive regular biennial or pre-employment healthcare checkups provided by the NHIS. This regular healthcare checkup includes anthropometric measurements, blood pressure, alcohol and smoking status, physical activity, and laboratory tests after overnight fasting, including serum glucose, total cholesterol, creatinine, liver function, and urinalysis [26–30]. Quality control procedures for laboratory tests were performed in accordance with the Korean Association of Laboratory Quality Control [27,30]. Past history, including stroke or coronary artery disease and lifestyle habits were collected by standardized self-report questionnaires. Additionally, the NHIS contains information on the patients’ demographics, medical use, transaction information, healthcare checkups, and claims [26–30].

This study was approved by the institutional review board (IRB) of the Catholic University of Korea (VC19ZESI0036). Informed consent for using their health information was exempted by the IRB. The study was conducted in compliance with the Declaration of Helsinki.

### Definition of NAFLD and comorbidities

**Fig 1:**
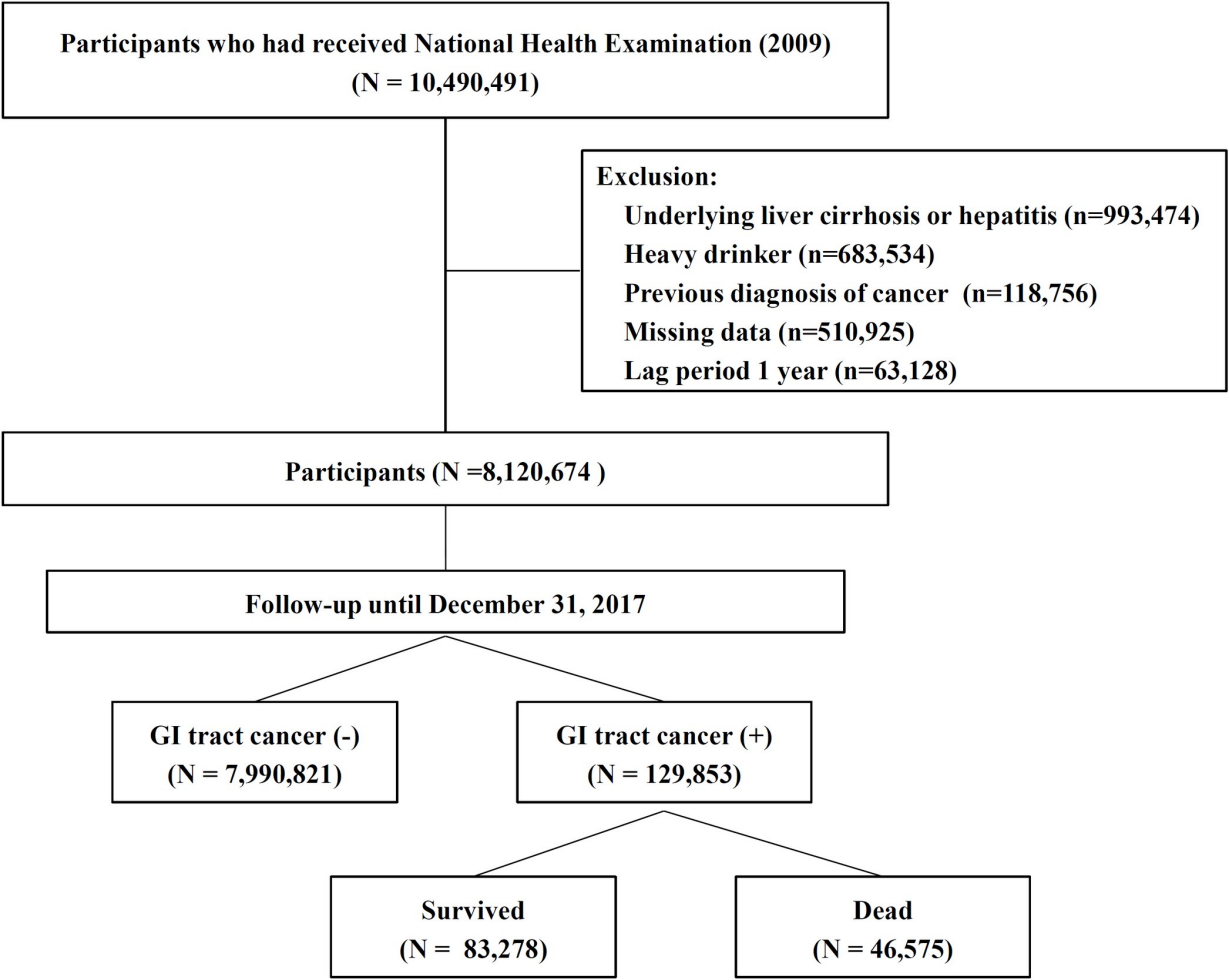
Flow diagram of study participants in the NHIS database with their reasons for inclusion and exclusion.

The inclusion criteria for the study population were as follows: 1) aged 20 years or older; 2) received healthcare checkups between January 1, 2009 and December 31, 2009; and 3) not diagnosed with any type of cancer before January 1, 2009 to exclude participants with a prior history of GI tract cancer (Fig 1). Comorbidity was defined using the International Classification of Diseases, 10^th^ revision (ICD-10) codes and the reimbursement code for confirmed cancer. Individuals with liver cirrhosis (ICD-10 codes of K703), any hepatitis (B15-19), heavy alcohol drinkers (≥30 g/day of alcohol use for men and women), or missing data for FLI were excluded.

Demographic characteristics were identified, including age, sex, body mass index (BMI), waist circumference (WC), and low socioeconomic status (lowest quartile of yearly income). Drinking status was defined as mild (<30 g/day) or non-drinking. Regular exercise was defined as engaging in high-intensity physical activity ≥1 time/week or moderate-intensity exercise ≥1/week [31]. Subjects were classified into obese and abdominal obesity groups when their BMI was ≥25 kg/m^2^ and WC was ≥90 cm in men and ≥85 cm in women, according to the criteria of the Asian-Pacific region [32,33].

After overnight fasting for at least 8 hours, blood specimens were collected and analyzed within 24 hours after transportation to the Central Testing Institute (Neodin Medical Institute, Seoul, Korea). The blood levels of glucose, creatinine, lipids, and liver enzymes [alanine transaminase (ALT), aspartate aminotransferase (AST), γ-glutamyl transferase (GGT)] were measured using a Hitachi 7600 automated chemistry analyzer (Hitachi, Tokyo, Japan).

We calculated the FLI according to an algorithm based on triglyceride level, BMI, WC and GGT and categorized the FLI groups as follows: < 30, 30–59, and ≥60 [24]:

$$FLI=(e^{0.953\times log\;\left(triglycerides\right))+0.139\times BMI+0.718\times log\;\left(GGT\right)+0.053\times waist\;circumference‐15.745})/\left(1+e^{0.953\times log\;\left(triglycerides\right)+0.139\times BMI+0.718\times log\;\left(GGT\right)+0.053\times waist\;circumference‐15.745}\right)\times 100.$$

Individuals with FLI≥60 in the absence of other causes of chronic liver disease were classified as having NAFLD [23,32]. Patients were classified as having type 2 diabetes mellitus (DM) when they had at least one service claim with a diagnosis of DM (E11-E14), either in outpatient or inpatient care, and were prescribed at least one antidiabetic drug (insulin or oral hypoglycemic agents) any time during a given year or had a fasting plasma glucose (FPG) level ≥126 mg/dL [27]. Hypertension was defined with the ICD-10 codes I10-I13 and I15 and treatment with anti-hypertensive agents or systolic or diastolic blood pressure ≥140 mmHg or ≥90 mmHg, respectively. Hyperlipidemia was defined as ICD-10 code E78 and treatment with lipid-lowering agents or total cholesterol ≥240 mg/dL [26,27,29–31]. Abdominal obesity was defined as waist circumference of ≥90 cm for men and ≥85 cm for women [32,33]. Chronic kidney disease (CKD) was defined as an estimated glomerular filtration rate (eGFR) <60 mL/min/1.73 m^2^, and eGFR was calculated from serum creatinine using the Modification of Diet in Renal Disease Study Group equation [34].

### Definition of primary outcome

The primary outcome was newly diagnosed esophageal, stomach, or colon cancer (ICD-10 codes of C16, C18-20, or C15, respectively), a reimbursement code for severe disease, or censoring for death. For all-cause mortality, those patients who developed any of the above three cancers and died of any cause during the follow-up were evaluated. All the subjects in this study were followed up from the index date until cancer diagnosis, death, withdrawal from NHIS, or the end of 2017

### Statistical analysis

Descriptive characteristics are presented as mean ± SD, median (IQR), numbers, or a frequency in percentage (%). The χ^2^ test was used to determine differences in the proportion of categorical variables, and ANOVA was used to evaluate differences between the means of continuous variables. Incidence rates of esophageal, stomach, and colorectal cancer are expressed as events per 1,000 patient-years. Participants were followed until the first diagnosis of GI tract cancer, death, or December 31, 2017.

Cox proportional hazards regression analysis was used to estimate the hazard ratios (HRs) and 95% confidence intervals (CIs) for the association between NAFLD and the development of GI tract cancer after adjustment for sex, age, BMI, drinking, smoking status, regular physical activity, income status, and comorbidities. Proportional hazards assumptions were evaluated by Schoenfeld residuals with the logarithm of the cumulative hazard function based on Kaplan-Meier estimates for the category of FLI. Given that mortality could compete with development of GI cancers, we performed a competing risk analysis using a sub-distribution hazards model. The multivariable-adjusted proportional hazards model was also used to evaluate the association between NAFLD and all-cause mortality. A two-sided *P* value <0.05 was considered statistically significant. Statistical analyses were performed using SAS version 9.4 (SAS Institute, Cary, NC).

## Results

### Clinical characteristics of the study population according to FLI category

From January 1, 2009 to December 31, 2009, 10,490,491 subjects had received a national health examination. After exclusion, 8,120,674 participants were included in this study (Fig 1). The mean age and BMI were 46.7 ± 14.1 years and 23.6 ± 3.2 kg/m^2^, respectively. A total of 52.1% were male. Among them, 936,159 adults (11.5%) were identified as having NAFLD (FLI ≥60). Compared to the subjects without NAFLD, subjects with NAFLD were more likely to be males, be current smokers, consume more alcohol, have a higher BMI, have a higher WC, and have hypertension or diabetes. In addition, individuals with NAFLD had higher fasting glucose, blood pressure, liver enzymes, and lipid levels compared to those in the FLI <60 group (Table 1).

**Table 1:** Baseline characteristics of study participants according to fatty liver index score category.

	Total population	Fatty liver index			
		<30	30–59	≥60	*P* value
N	8,120,674	5,348,282	1,836,233	936,159	
Age (years)	46.7 ± 14.1	45.5 ± 14.4	50.0 ± 13.5	47.4 ± 12.7	< .0001
Age (years)					< .0001
< 40	2,642,440 (32.5)	1,906,272 (35.6)	450,197 (24.5)	285,971 (30.6)	
40–64	4,452,193 (54.9)	2,821,739 (52.8)	1,085,420 (59.1)	545,034 (58.2)	
≥ 65	1,026,041 (12.6)	620,271 (11.6)	300,616 (16.4)	105,154 (11.2)	
Sex					< .0001
Male (%)	4,234,418 (52.1)	2,193,432 (41.0)	1,277,293 (69.6)	763,693 (81.6)	
Female (%)	3,886,256 (47.9)	3,154,850 (59.0)	558,940 (30.4)	172,466 (18.4)	
BMI (kg/m^2^)	23.6 ± 3.2	22.2 ± 2.4	25.5 ± 2.2	27.9 ± 3.0	< .0001
WC (cm)	79.8 ± 9.1	75.5 ± 7.0	85.9 ± 5.4	92.1 ± 6.8	< .0001
Diabetes (yes)	642,417 (7.91)	255,004 (4.77)	220,559 (12.0)	166,854 (17.8)	< .0001
Hypertension (yes)	1,972,622 (24.3)	919,994 (17.2)	644,793 (35.1)	407,835 (43.6)	< .0001
Dyslipidemia (yes)	1,435,523 (17.7)	651,749 (12.2)	465,358 (25.3)	318,416 (34.0)	< .0001
CKD (yes)	476,036 (5.9)	287,831 (5.4)	127,987 (7.0)	60,218 (6.43)	< .0001
Smoking status					< .0001
Non-smokier	5,050,964 (62.2)	3,767,603 (70.5)	922,267 (50.2)	361,094 (38.6)	
Ex-smoker	1,085,310 (13.4)	554,858 (10.4)	343,155 (18.7)	187,297 (20.0)	
Current smoker	1,984,400 (24.4)	1,025,821 (19.2)	570,811 (31.1)	387,768 (41.4)	
Alcohol consumption					< .0001
Non	4,402,965 (54.2)	3,158,256 (59.1)	893,769 (48.7)	350,940 (37.5)	
Mild	3,717,709 (45.8)	2,190,026 (41.0)	942,464 (51.3)	585,219 (62.5)	
Lower physical activity (yes) ^a^	4,155,923 (51.2)	2,677,738 (20.1)	969,368 (52.8)	508,817 (54.4)	< .0001
Income (Q1)	2,187,148 (26.9)	1,528,844 (28.6)	440,320 (24.0)	2,179,84 (23.3)	< .0001
SBP (mm Hg)	122.0 ± 14.9	119.1 ± 14.3	126.5 ± 14.3	129.8 ± 14.5	< .0001
DBP (mm Hg)	76.0 ± 9.94	74.1 ± 9.5	78.8 ± 9.5	81.4 ± 9.9	< .0001
Fasting glucose (mg/dL)	96.5 ± 22.1	93.2 ± 18.0	100.7 ± 25.1	106.7 ± 31.4	< .0001
TC (mg/dL)	195.2 ± 36.5	189.1 ± 34.4	203.9 ± 36.6	212.5 ± 38.8	< .0001
HDL-C (mg/dL)	55.4 ± 18.8	58.0 ± 17.6	51.3 ± 19.8	48.8 ± 20.0	< .0001
LDL-C (mg/dL)	113.9 ± 33.5	112.0 ± 31.7	119.5 ± 35.0	114.0 ± 38.7	< .0001
eGFR (ml/min/1.73 m^2^)	88.3 ± 44.1	89.3 ± 43.4	86.3 ± 44.3	87.0 ± 47.9	< .0001
ALT (IU/L)*	20 (14–28)	17 (13–22)	25 (19–35)	36 (25–52)	< .0001
AST (IU/L)*	22 (18–27)	21 (18–25)	24 (20–29)	29 (23–37)	< .0001
GGT (IU/L)*	22 (15–36)	18 (13–24)	34 (24–49)	60 (39–96)	< .0001
Triglyceride (mg/dL)*	107 (73–160)	86 (63–117)	153 (116–203)	224 (164–312)	< .0001

BMI, body mass index; WC, waist circumference; CKD, chronic kidney disease; Q1, Lower quintile of yearly income; SBP, systolic blood pressure; DBP, diastolic blood pressure; TC, total cholesterol, HDL-C, high density lipoprotein cholesterol; LDL-C, low density lipoprotein cholesterol; eGFR, estimated glomerular filtration rate; AST, aspartate aminotransferase; ALT, alanine transaminase; GGT, gamma-glutamyltransferase.

Variables are expressed as mean ± SD or n (%).

^a^Persons who did not perform high intensity or moderate intensity of activity ≥ 1/week;

*median (IQR)

During the follow-up period of 7.2 years, 3,792 esophageal, 57,292 stomach and 68,769 colorectal cancer cases were newly identified, for incidence rates of 0.064, 0.97, and 1.16 per 1,000 person-years, respectively. The age- and sex-adjusted HRs for subjects with NAFLD were 1.26 (95% CI 1.16–1.38) for esophageal, 1.18 (95% CI 1.15–1.21) for stomach, and 1.30 (95% CI 1.27–1.33) for colorectal cancer, respectively, compared to those with FLI <30 (Table 2). This significant association persisted after further adjustment for BMI, smoking and drinking habits, regular exercise, low socioeconomic status, and the presence of diabetes. NAFLD (FLI ≥60) was significantly associated with the development of esophageal (HR 2.10, 95% CI 1.88–2.35), stomach (HR 1.18, 95% CI 1.14–1.22), and colon cancer (HR, 1.23, 95% CI 1.19–1.26) (Table 2). A competing risk analysis that included mortality as a competing risk also resulted in similar outcome as the main results (S1 Table).

**Table 2:** Incidence rate and hazard ratios for the risk of esophageal, stomach and colorectal cancers.

Fatty liver index		Event	IR*	Model 1	Model 2
Composite outcome					
	0-<30	66,688	1.72	1 (ref)	1 (ref)
	30–59	35,346	2.67	1.11 (1.10, 1.13)	1.11 (1.10, 1.13)
	≥60	17,769	2.64	1.24 (1.22, 1.27)	1.22 (1.20, 1.25)
Esophageal cancer					
	0-<30	2,031	0.05	1 (ref)	1 (ref)
	30–59	1,088	0.08	0.94 (0.87, 1.01)	1.30 (1.19, 1.41)
	≥60	673	0.10	1.26 (1.16, 1.38)	2.10 (1.88, 2.35)
Stomach cancer					
	0-<30	31,606	0.81	1 (ref)	1 (ref)
	30–59	17,132	1.29	1.08 (1.06, 1.10)	1.09 (1.06, 1.11)
	≥	8,554	1.27	1.18 (1.15, 1.21)	1.18 (1.14, 1.22)
Colorectal cancer					
	0-<30	38,535	0.99	1 (ref)	1 (ref)
	30–59	20,150	1.52	1.15 (1.12, 1.17)	1.12 (1.09, 1.14)
	≥60	10,084	1.49	1.30 (1.27, 1.33)	1.23 (1.19, 1.26)

*, incidence rate of each cancer (events/1,000 patient-year). Model 1: age, sex, Model 2: age, sex, smoking status, drinking habit, regular exercise, yearly income (lowest Q1), BMI, diabetes

### Subgroup analyses by diabetes or obesity status

**Fig 2:**
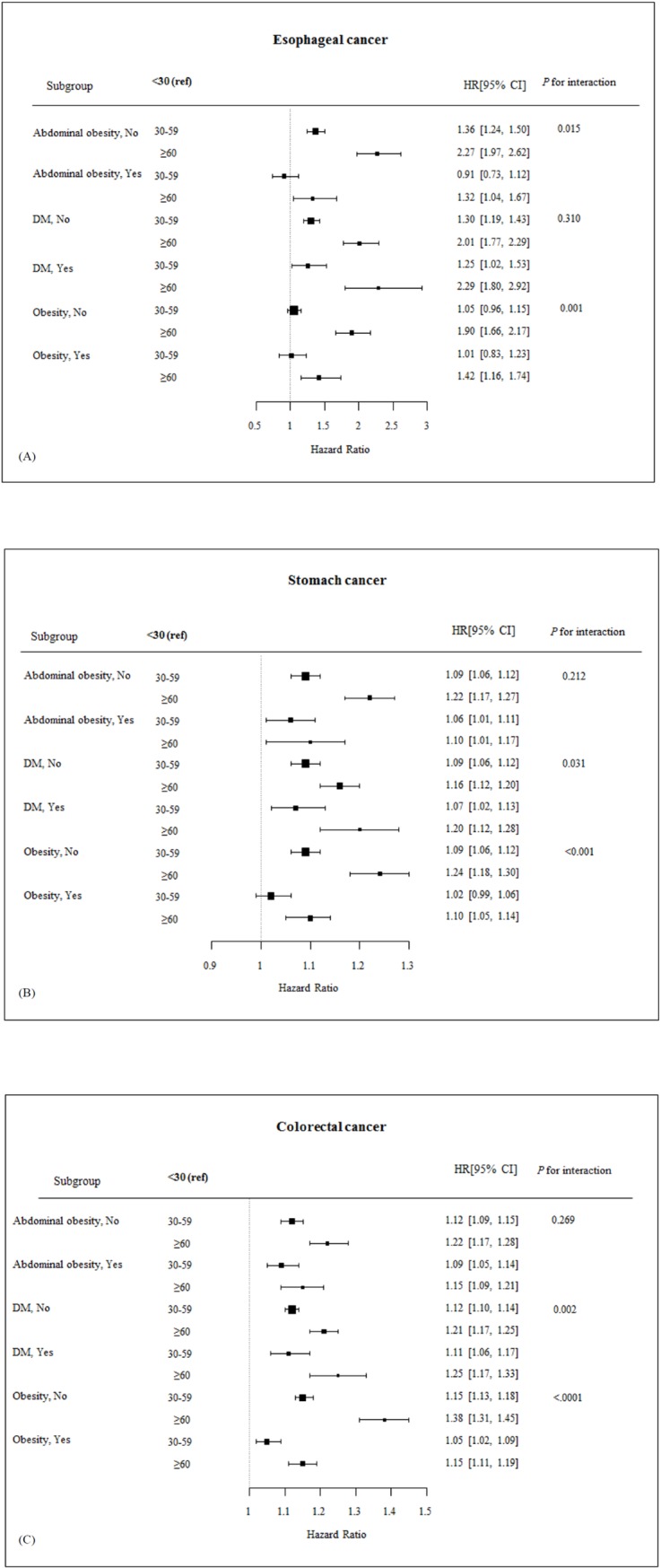
Forest plots for the association between FLI and esophageal (A), stomach (B) and colorectal cancer (C) in subgroups. All HRs adjusted for covariates including age, sex, smoking status, drinking habit, regular exercise, yearly income, BMI, and diabetes.

We performed subgroup analyses according to the presence or absence of diabetes, general obesity, and abdominal obesity (Fig 2). With increasing FLI category, HRs for developing esophageal, stomach, and colorectal cancers showed increasing trends. The associations between FLI and incident stomach and colorectal cancers tended to be stronger in patients with DM than in those without DM (*P* for interaction, 0.031 and 0.002, respectively), although there was no noticeable difference in HR. This trend was not found in subjects with esophageal cancer (*P* for interaction = 0.301). Interestingly, the associations between FLI and all three cancers were stronger in patients without obesity than in those with obesity (*P* for interaction < 0.001 for all).

We further analyzed our data to investigate the association between BMI and the development of GI cancers. Thus, BMI was stratified into normal (BMI <23 kg/m^2^), overweight (23-<25 kg/m^2^), and obese (≥25 kg/m^2^) populations across each GI cancer. In all BMI categories, patients with NAFLD showed higher HRs for all three GI cancers. In addition, compared to the obese-NAFLD group, the HRs of normal weight-NAFLD showed significantly higher in all three cancers (*P* value for interaction, < 0.0001 for all 3 cancers). We suggested that the association between NAFLD and GI cancers is more remarkable in a non-obese population (S2 Table).

### Effects of individual components of the FLI on the development of each cancer

The incidence rates and HRs of esophageal, stomach, and colorectal cancers according to the individual components of FLI (BMI cut-off, abdominal obesity, triglyceride level (*≥*150 mg/dL or lipid-lowering agent use) and the upper 25% of GGT are listed in Table 3. Obesity (WC, BMI *≥*25.0 kg/m^2^) was associated with increased risk of stomach and colorectal cancers and decreased risk of esophageal cancer. The GGT upper quartile (*≥*36 IU/mL) was associated with an increased risk of esophageal, stomach, and colorectal cancers. No association was observed with serum TG levels.

**Table 3:** Incidence rate and hazard ratios for the risk of esophageal, stomach and colorectal cancers by each component of fatty liver index.

	Esophagus cancer		Stomach cancer		Colorectal cancer	
	IR*	model	IR*	model	IR*	model
Triglyceride ≥150 mg/dL or lipid lowering treatment						
No	0.06	1 (ref)	0.84	1 (ref)	1.01	1 (ref)
Yes	0.08	1.03 (0.96,1.10)	1.23	0.99 (0.97,1.01)	1.49	1.01 (0.998,1.03)
BMI (≥25 kg/m^2^)						
No	0.07	1 (ref)	0.92	1 (ref)	1.08	1 (ref)
Yes	0.05	0.72 (0.66,0.77)	1.09	1.03 (1.01,1.05)	1.36	1.09 (1.08,1.11)
WC (≥90 cm in men, ≥ 85 cm in women)						
No	0.06	1 (ref)	0.88	1 (ref)	1.04	1 (ref)
Yes	0.08	0.91 (0.84,0.98)	1.37	1.04 (1.02,1.06)	1.73	1.13 (1.11,1.14)
GGT (upper quartile, ≥ 36 IU/L)						
No	0.05	1 (ref)	0.88	1 (ref)	1.08	1 (ref)
Yes	0.12	1.82 (1.70,1.95)	1.26	1.11 (1.09,1.13)	1.42	1.15 (1.13,1.17)

*, incidence rate of each cancer (events/1,000 patient-year). Model: age, sex, smoking status, drinking habit, regular exercise, yearly income (lowest Q1), BMI, diabetes

### All-cause mortality and FLI category

During the observation period, 46,575 subjects (0.6% of the total study population, 35.9% of newly diagnosed GI tract cancer patients) died. More subjects with NAFLD died than those without NAFLD in all 3 cancer groups. The all-cause mortality rates were significantly higher in the FLI *≥*60 group compared to the FLI <60 group (24.3 vs. 26.0 in esophageal cancer, 6.75 vs. 6.12 in stomach cancer, and 7.06 vs. 7.06 per 1,000 patient-years in colorectal cancer, respectively (Table 4). Additionally, NAFLD (FLI score *≥*60) was associated with an increased risk of all-cause mortality in patients with esophageal cancer (HR 1.46, 95% CI 1.28–1.67), stomach cancer (HR 1.26, 95% CI 1.18–1.34), and colorectal cancer (HR 1.16, 95% CI 1.10–1.22) (Table 4).

**Table 4:** Mortality rate and hazard ratio for all-cause mortality of each cancer according to NAFLD.

Fatty liver index		Events	Mortality rate (per 1,000 person-year)	HR (95% CI)
Esophagus cancer				
	< 60	1,531	24.32	1 (ref)
	≥ 60	332	26.03	1.46 (1.28, 1.67)
Stomach cancer				
	< 60	10,019	6.75	1 (ref)
	≥ 60	1,576	6.12	1.26 (1.18, 1.34)
Colorectal cancer				
	< 60	12,297	7.06	1 (ref)
	≥ 60	20,820	7.06	1.16 (1.10, 1.22)

**Fig 3:**
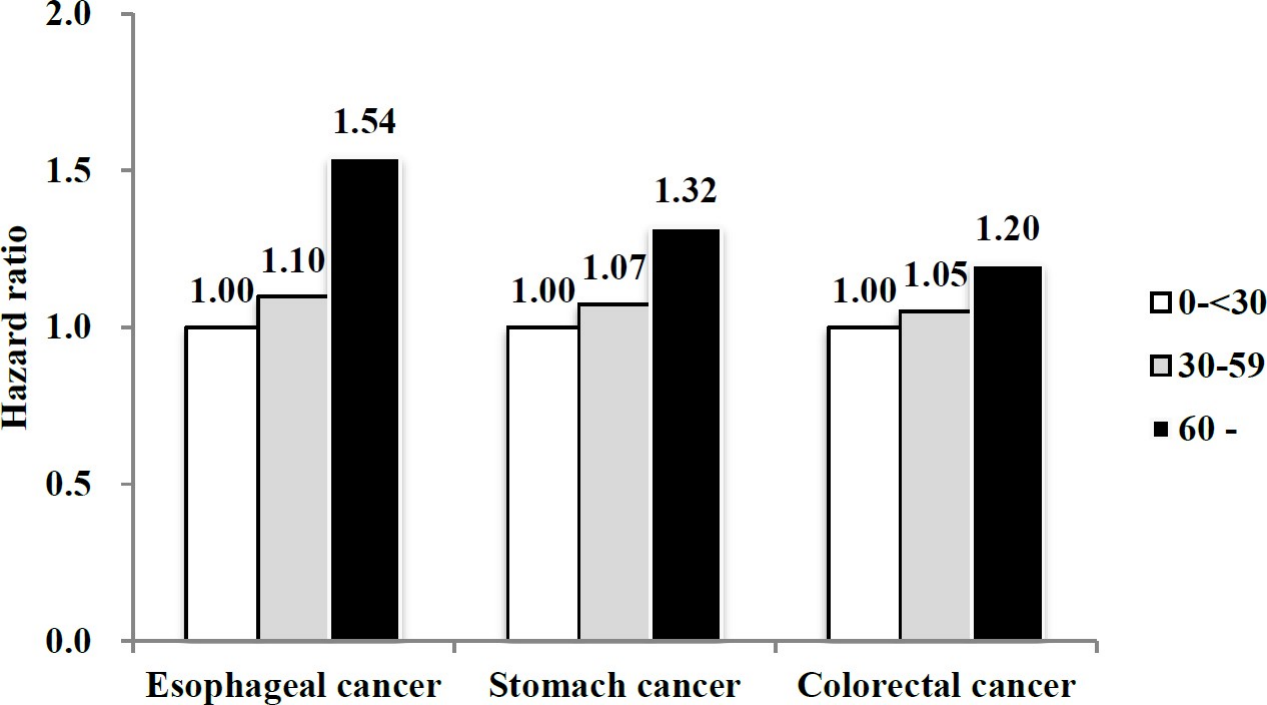
Hazard ratios for all-cause mortality according to FLI category in patients with esophageal, stomach, and colorectal cancer. Data are HRs (95% CI). All HRs adjusted for covariates including age, sex, smoking status, drinking habit, regular exercise, yearly income (lowest Q1), BMI, and diabetes.

When the FLI was classified into 3 categories (<30, 30–59, *≥*60), the risk of all-cause mortality increased with increasing category of FLI in all the cancer groups (*P* for trend <0.0001 for esophageal, stomach, and colorectal cancer groups, Fig 3).

## Discussion

In this large, nationally representative, population-based cohort analysis using a national database of health insurance claims in Korea, we demonstrated a significant positive association between NAFLD and future development of GI tract malignancy, including esophageal, stomach, and colon cancers, in the general population. Subjects with NAFLD had an approximately 1.2-two-fold increased risk of GI tract cancers. To the best of our knowledge, the present study is the first evidence of NAFLD as a risk factor for extrahepatic GI tract malignancy in an adult Asian population.

The association between NAFLD and some GI cancers has also been reported previously. NAFLD was associated with a high prevalence of colorectal adenomatous polyp, colorectal adenoma, and colorectal cancer [14,35–38]. In a prospective study, the incidence of colon adenoma development was increased by 45% in patients with NAFLD [39,40]. However, the presence of NAFLD had no influence on the progression or recurrence of colorectal cancer [36,41]. Compared to colorectal neoplasms, the association between NAFLD and other GI tract cancers is less proven. According to a population-based retrospective cohort study from the Taiwan National Health Insurance (NHI) program database, patients with nonalcoholic cirrhosis (NAC) showed significantly higher risks of digestive tract cancers compared with those without NAC [42]. Studies on the association between NAFLD and stomach cancer are more limited. Only one observational study from a single center in Turkey showed that the prevalence of NAFLD was higher in subjects with gastric cancers [43]. In a study from a medical records review, the rate of gastric cancer was significantly higher in patients with alcoholic liver disease than in healthy controls [44].

In this study, we calculated the FLI score in the general population using a national health examination database and measured the relationship between NAFLD (FLI ≥60) and esophageal, stomach, and colorectal cancers, which are the most prevalent GI cancers in the Korean population. After adjustment for multiple clinical covariates, NAFLD, defined as an FLI score of ≥60, was associated with increased risk of incident esophageal, stomach and colorectal cancers. In subgroup analyses, increased risk of incident esophageal, stomach, and colorectal cancer among those with NAFLD was seen, mainly in non-obese subjects. In addition, the increased risk of incident stomach and colorectal cancers among subjects with NAFLD tended to be higher in patients with DM compared to the nondiabetic population. We suggest that the influence of NAFLD (defined as FLI score) on incident esophageal, stomach, and colorectal cancers might be more prominent in the non-obese population. Therefore, non-obese individuals with NAFLD might be a target population for screening for these three cancers. When the four components of FLI were subdivided, we found that obesity (WC and BMI criteria) showed opposite relationships with incident esophageal cancer vs. stomach or colorectal cancer. The HRs for stomach and colorectal cancers were increased in the obese population, but the HR for esophageal cancer was decreased. Several epidemiologic studies suggest the association between lower BMI and esophageal cancer [45,46]. Poor diet, commonly observed in individuals who are underweight, can lead to malnutrition and have been implicated as high-risk factors for esophageal cancer, especially in Asian populations [45,46].

Interestingly, the highest quartile of GGT level was significantly associated with the development of all three cancers. *γ*-Glutamyltransferase (GGT) is a marker for hepatic injury and alcohol consumption and plays a central role in the homeostasis of the antioxidant glutathione (GSH). The expression of GGT has been shown to be upregulated after oxidative stress, but the signaling pathways implicated remain poorly characterized [47]. Previous studies have reported on the associations of serum GGT level with the risk of cancer [48]. Several potential mechanisms have been postulated for the relationship between GGT and cancer: As essential parts of the cellular defense apparatus, GGT and GSH combat oxidative stress. Increased GGT has been regarded as a marker of exposure to certain carcinogens. GGT levels can be affected by environmental and lifestyle factors (such as diet, smoking, and drinking) and genetic regulation [48]. Based on a retrospective study performed in 447 patients with esophageal squamous cell carcinoma, higher GGT might predict worse overall survival than normal GGT [46]. A large study of new cancer cases, which occurred among 1,662,087 Koreans from the National Health Insurance Service database during 1995 and 1998 who were followed up for 17 years, showed that an elevated serum level of GGT was independently linked with the risk of various tumors, such as colorectal, stomach, lung and bile duct cancer [49]. Clinical data from 8,388,256 Korean individuals aged 40 years and over who received national healthcare check-ups in 2007 and 2008 showed an increased risk of esophageal cancer in subjects with serum GGT values >18 IU/L, regardless of age, sex, smoking status, or alcohol consumption [50].

The pathophysiological mechanisms resulting in GI tract malignancy in NAFLD are not fully understood. Recently, putative mechanisms linking NAFLD and extrahepatic neoplasms have been suggested, such as insulin resistance, dysfunctional adipose tissue, chronic inflammation, and alterations of gut microbiota [51]. One possible mechanism explaining the increased risk of colorectal neoplasia in NAFLD patients with advanced fibrosis is a pro-inflammatory and insulin-resistant condition that elevates serum insulin and insulin-like growth factor-1 (IGF-1), which in turn may promote growth and anti-apoptosis of colorectal neoplasia [25,35,52]. Some studies have suggested that tumor necrosis factor-alpha, interleukin-6, adiponectin, leptin and other pro-inflammatory cytokines play a role in the development of colorectal neoplasia, which can be altered in NASH as well [51]. Obesity is also a well-recognized, major risk factor in the development of various cancers, such as colorectal, esophageal, liver, cardia gastric, and pancreatic cancers [51,53]. Interestingly, the relationship between visceral obesity and esophageal adenocarcinoma was independent of gastro-esophageal reflux disease and was possibly mediated by insulin resistance and chronic inflammation [51].

Importantly, compared to patients without NAFLD, patients with NAFLD with incident esophageal, stomach, or colorectal cancer showed significantly increased all-cause mortality during the observation period in this study. Given the increased mortality in cancer patients with NAFLD, adipocytokines might link obesity-related disorders with neoplasm development both intra- and extrahepatically [2]. The steatotic and inflamed liver may secrete growth-promoting factors into the systemic circulation [53]. NAFLD-derived plasminogen activator inhibitor 1, vascular endothelial growth factor (VEGF) or angiopoietin may be involved in metastasis and thus cancer progression [54].

NAFLD should be diagnosed based on imaging or pathologic findings. However, routine abdominal ultrasonography and liver biopsy cannot be performed routinely in the general population. Instead, some biomarkers, such as the fatty liver index (FLI), calculated using 4 variables (BMI, WC, TG, and GGT), are readily available, simple, and noninvasive parameters for predicting NAFLD. The accuracy of the FLI in comparison with the US method for detecting and quantifying hepatic steatosis has been validated in several other studies. The validation of the FLI for NAFLD was conducted in a large number of Chinese adults aged ≥40 years [22] and in a Western population-based study [21]. Overall, to rule in fatty liver, a cutoff of ≥30 had a sensitivity of 62% and a specificity of 88% for predicting the presence of NAFLD. FLI ≥60 was 60.4% and 82.3%, respectively [23]. High FLI (≥30) was associated with an increased risk of colorectal adenoma in the Asian population [24,25].

Although the strengths of this study included a large nationwide population, there are some limitations to this analysis. It relied only on claim data; therefore, we could not obtain clinical information on histology, imaging, or treatment of newly diagnosed GI cancers. We did not have information on the severity of liver disease in terms of inflammation and fibrosis because liver biopsies would be unethical in a population-based study. The cause of death could not be confirmed by claim data. During calculation of FLI, some subjects had lipid-lowering medications that influencing TG level. The time interval between the initiation of lipid-lowering agent and measurement of FLI was not considered in this analysis. Finally, the Korean population shows disparities in the prevalence, location, and shape characteristics of colorectal neoplasia compared to Western countries [55]. The generalizability of this study to other ethnic groups needs to be confirmed.

Despite these limitations, the major strength of this study was that the data, including anthropometric, clinical information, and baseline laboratory results, were based on a nationwide Korean population covering nearly 100% of the Korean adult population, so they provide evidence regarding real-world clinical practice.

## Conclusions

NAFLD, defined using FLI, was a good predictive indicator for GI tract malignancy in the general population. In addition to liver cirrhosis or hepatocellular carcinoma, additional active surveillance or screening of GI tract cancers is needed in subjects with NAFLD. Future research should be conducted to define the mechanism linking NAFLD and GI malignancy. In addition, strategies to improve NAFLD might reduce the development of GI tract cancer or prevent cancer-associated mortality. Prompt interventions, such as lifestyle modification, should be provided in this high-risk population.

## Supporting information

S1 Table Competing risk analysis including mortality as a competing risk. (DOCX)

S2 Table Risk and HRs of GI cancers stratified by BMI across the FLI score category. (DOCX)
