# Supplementary material for: The association between nonalcoholic fatty liver disease and esophageal, stomach, or colorectal cancer: National population-based cohort study
Source: PLoS One. 2020 Jan 24;15(1):e0226351. doi: 10.1371/journal.pone.0226351 (PMC6980645; doi:10.1371/journal.pone.0226351)
Supplement: S1 Table — (DOCX) [file pone.0226351.s001.docx]

**S1 Table. Competing risk analysis including mortality as a competing risk.**

| FLI Group | Composite | Esophageal cancer | Stomach  cancer | Colorectal  cancer |
| --- | --- | --- | --- | --- |
| Model 1 |  |  |  |  |
| 0-<30 | 1 (ref) | 1 (ref) | 1 (ref) | 1 (ref) |
| 30-59 | 1.13 (1.12, 1.15) | 0.96 (0.89, 1.03) | 1.10 (1.08, 1.12) | 1.17 (1.15, 1.19) |
| ≥60 | 1.26 (1.24, 1.28) | 1.28 (1.17, 1.40) | 1.19 (1.17, 1.22) | 1.31 (1.29, 1.34) |
| Model 2 |  |  |  |  |
| 0-<30 | 1 (ref) | 1 (ref) | 1 (ref) | 1 (ref) |
| 30-59 | 1.11 (1.09, 1.12) | 1.28 (1.17, 1.40) | 1.08 (1.06, 1.11) | 1.12 (1.09,1.14) |
| ≥60 | 1.20 (1.17, 1.23) | 2.01 (1.79, 2.26) | 1.15 (1.12, 1.19) | 1.20 (1.17,1.24) |

Model 1; age, sex

Model 2; age, sex, smoking status, drinking habit, regular exercise, yearly income (lowest Q1), BMI, diabetes
