# Supplementary material for: The association between nonalcoholic fatty liver disease and esophageal, stomach, or colorectal cancer: National population-based cohort study
Source: PLoS One. 2020 Jan 24;15(1):e0226351. doi: 10.1371/journal.pone.0226351 (PMC6980645; doi:10.1371/journal.pone.0226351)
Supplement: S2 Table — (DOCX) [file pone.0226351.s002.docx]

**S2 Table. Risk and HRs of GI cancers stratified by body mass index (BMI) across the fatty liver index (FLI) score category.**

|  | FLI score | Composite | | | Esophageal cancer | | | Stomach cancer | | | Colorectal cancer | | |
| --- | --- | --- | --- | --- | --- | --- | --- | --- | --- | --- | --- | --- | --- |
| BMI | | IR | Model 1 | Model 2 | IR | Model 1 | Model 2 | IR | Model 1 | Model 2 | IR | Model 1 | Model 2 |
| <23 kg/m^2^ | |  |  |  |  |  |  |  |  |  |  |  |  |
|  | <30 | 1.62 | 1 (ref) | 1 (ref) | 0.06 | 1 (ref) | 1 (ref) | 0.78 | 1 (ref) | 1 (ref) | 0.92 | 1 (ref) | 1 (ref) |
|  | 30-59 | 3.39 | 1.29  (1.25,1.32) | 1.23  (1.19,1.26) | 0.19 | 1.42  (1.25, 1.61) | 1.43  (1.25,1.63) | 1.66 | 1.21  (1.16, 1.26) | 1.16  (1.11, 1.21) | 1.82 | 1.33  (1.28, 1.39) | 1.26  (1.21, 1.31) |
|  | ≥60 | 4.54 | 1.64  (1.53,1.75) |  | 0.51 | 3.45  (2.81, 4.23) | 3.05  (2.47,3.76) | 2.12 | 1.43  (1.30, 1.58) | 1.33  (1.20, 1.47) | 2.33 | 1.66  (1.51, 1.82) | 1.50  (1.37, 1.65) |
| 23-<25 kg/m^2^ | |  |  |  |  |  |  |  |  |  |  |  |  |
|  | <30 | 1.91 | 1 (ref) | 1 (ref) | 0.043 | 1 (ref) | 1 (ref) | 0.89 | 1 (ref) | 1 (ref) | 1.12 | 1 (ref) | 1 (ref) |
|  | 30-59 | 2.75 | 1.11  (1.08,1.14) | 1.08  (1.05,1.10) | 0.099 | 1.30  (1.13,1.49) | 1.23  (1.07, 1.42) | 1.37 | 1.11  (1.07, 1.14) | 1.07  (1.04, 1.11) | 1.51 | 1.11  (1.08, 1.15) | 1.08  (1.04, 1.11) |
|  | ≥60 | 3.34 | 1.40  (1.34,1.46) | 1.30  (1.25,1.36) | 0.120 | 2.53  (2.10, 3.05) | 2.21  (1.82, 2.69) | 1.63 | 1.32  (1.24, 1.40) | 1.24  (1.16, 1.32) | 1.81 | 1.41  (1.33, 1.49) | 1.30  (1.23, 1.38) |
| ≥25 kg/m^2^ | |  |  |  |  |  |  |  |  |  |  |  |  |
|  | <30 | 1.85 | 1 (ref) | 1 (ref) | 0.0284 | 1 (ref) | 1 (ref) | 0.82 | 1 (ref) | 1 (ref) | 1.13 | 1 (ref) | 1 (ref) |
|  | 30-59 | 2.49 | 1.06  (1.03,1.08) | 1.04  (1.01,1.07) | 0.051 | 1.06  (0.87,1.29) | 1.05  (0.86,1.28) | 1.17 | 1.04  (1.01, 1.08) | 1.03  (0.99, 1.07) | 1.46 | 1.07  (1.03, 1.10) | 1.05  (1.01, 1.08) |
|  | ≥60 | 2.48 | 1.18  (1.15,1.22) | 1.14  (1.10,1.18) | 0.072 | 1.58  (1.30,1.92) | 1.56  (1.25, 1.93) | 1.19 | 1.15  (1.10, 1.20) | 1.11  (1.06, 1.17) | 1.42 | 1.20  (1.15, 1.24) | 1.14  (1.09, 1.19) |
| *P* value for interaction | |  | <.0001 | <.0001 |  | <.0001 | <.0001 |  | <0.0001 | <0.0001 |  | <0.0001 | <0.0001 |

BMI, body mass index; FLI, fatty liver index

IR, incidence rate of each cancer (events per 1,000 person-years).

Model 1: age, sex

Model 2: age, sex, smoking status, drinking habit, regular exercise, yearly income (lowest Q1), BMI, diabetes
